# Tunable Photonic Applications Based on Electrochemical Mechanisms

**DOI:** 10.1002/nap2.70062

**Published:** 2026-04-09

**Authors:** Hyo Eun Jeong, Ji‐Eun Yeo, Hyeon‐Ho Jeong, Young Min Song

**Affiliations:** ^1^ School of Electrical Engineering Korea Advanced Institute of Science and Technology Daejeon South Korea; ^2^ GIST InnoCORE AI‐Nano Convergence Institute for Early Detection of Neurodegenerative Diseases Gwangju Institute of Science and Technology Gwangju South Korea; ^3^ School of Electrical Engineering and Computer Science Gwangju Institute of Science and Technology Gwangju South Korea

**Keywords:** colorimetric sensor, electrochemical doping, electrochromic display, nanotechnology, optical memory, structural color, tunable photonics

## Abstract

Reflective displays offer key advantages such as high energy efficiency, eye comfort, and excellent visibility, making them attractive for a wide range of applications. Recently, tunable photonics has focused on integrating electrochromic (EC) materials with optical resonant structures, enabling dynamic control of reflection spectra and intensity through enhanced light–matter interaction. Here, this review provides an overview of recent progress in electrochemically driven dynamic coloration devices, systematically categorized according to their tuning mechanisms and photonic design within the visible spectral range. We outline energy‐efficient and reconfigurable optical modulation strategies enabled by EC materials that operate through ionic insertion, redox reactions, or reversible plating/stripping of ionic metals. Furthermore, by bridging electrochemistry and photonic structures, we highlight recent progress, challenges, and emerging applications in reflective displays, colorimetric sensing, optical memory, and active holography. This review concludes by addressing the key challenges and future directions of this rapidly evolving field, highlighting the scientific and technological significance of electrochemical photonics as a foundation for next‐generation tunable optical systems.

## Graphical abstract

Electrochromic materials integrated into wearable platforms enable voltage‐driven, reversible color modulation for sensing and display functionalities. Smart eyewear, electrochromic textiles, skin‐mounted devices, and biosensors demonstrate coupling between electrochemical optical control and adaptive feedback. Such multifunctional systems advance flexible, low‐power, and interactive wearable technologies for next‐generation human–machine interfaces.

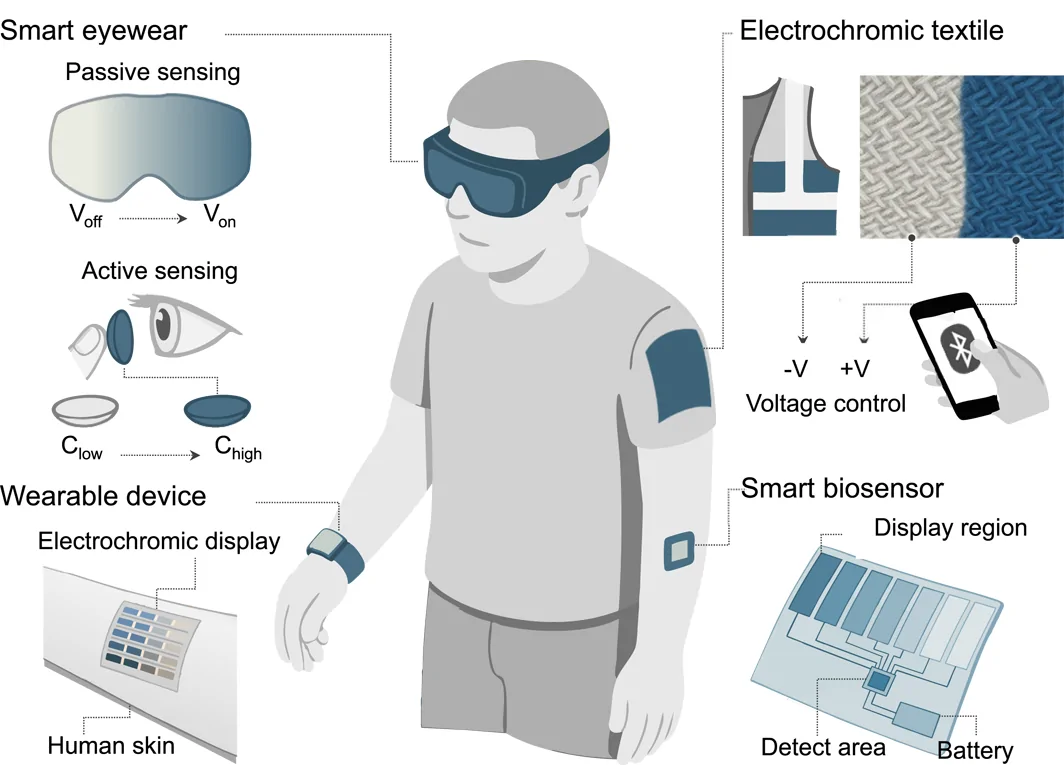

## 1 Introduction

In the modern data‐driven era, individuals continuously generate and exchange vast amounts of information, and dynamic display technologies have become vital interfaces for visual communication and human–device interaction. Traditionally, pigment‐based coloration has been widely employed due to its simplicity and low fabrication cost. However, this approach suffers from limitations in long‐term chemical stability and environmental sustainability, and its fixed optical absorption spectrum restricts its versatility for advanced applications. At present, commercial displays are dominated by emissive technologies, such as liquid crystal displays (LCDs) and organic light‐emitting diodes (OLEDs), which offer high optical performance, resolution, contrast ratio, wide viewing angle, and fast response speed, supported by mature fabrication processes. Despite these advantages, emissive displays inherently exhibit eye fatigue during prolonged operation, high power consumption due to the backlight unit, and limited visibility under bright ambient conditions.

As an alternative, reflective displays have attracted increasing attention. By forming images through the reflection of ambient light, these systems reduce visual strain and improve energy efficiency. Moreover, with the development of structural color technologies, reflective displays provide an environmentally sustainable and chemically stable approach to color generation [1, 2]. Combining high reflectance, fast response, and dynamic color tunability, they hold significant promise as next‐generation low‐power display and optoelectronic platforms (Figure 1A). Liquid crystal (LC)‐based [3, 4, 5] and electrophoretic display (EPD) [6, 7] technologies, widely used in electronic paper (E‐paper), generate images by modulating the active layer under an applied electric field. These systems achieve high contrast and visibility by modulating reflected ambient light; however, most commercial devices are restricted to using subpixel architectures or predefined colors determined by the embedded pigment particles during fabrication due to material and structural constraints [8]. In addition, their relatively high driving voltage (∼10 V) and slow response speed of several seconds hinder practical implementation in advanced display platforms [9]. Reflective spectra are inherently constrained by the maximum achievable reflectance, which limits both brightness and color quality. Optimizing chromaticity is insufficient, as high reflectivity and fast optical response must be achieved simultaneously for high‐performance electronic paper. In modern days, recent studies have explored highly reflective optical interfaces based on thin‐film cavities [10], plasmonic nanostructures [11, 12, 13], and metasurface architectures [14, 15, 16, 17]. However, conventional RGB subpixel configurations occupy only 1/3 of the reflective area for each color, leading to a significant reduction in overall reflectance, even when theoretical 100% reflection is achieved. To address this issue, display technologies are evolving from fixed‐color subpixel arrays toward optically tunable reflective monopixel architectures capable of dynamic color modulation within a single pixel (Figure 1B). Recent reviews have extensively summarized electrochromic materials and device technologies from materials chemistry and electrochemical performance perspectives, particularly for displays, smart windows, and related optoelectronic devices [18, 19, 20]. More recently, electrochromics have also been discussed as electrochemically tunable optical media [21]; however, a system‐level photonic perspective linking these materials with functional nanophotonic architectures for sensing, imaging, and spectrally engineered optical systems remains comparatively underexplored. Tunable monopixels rely on active materials whose optical properties can be controlled by electrical stimuli. Among them, electrochemically driven materials demonstrate strong potential for practical applications due to their low operating voltage, mechanical flexibility, and compatibility with flexible substrates.

**FIGURE 1:**
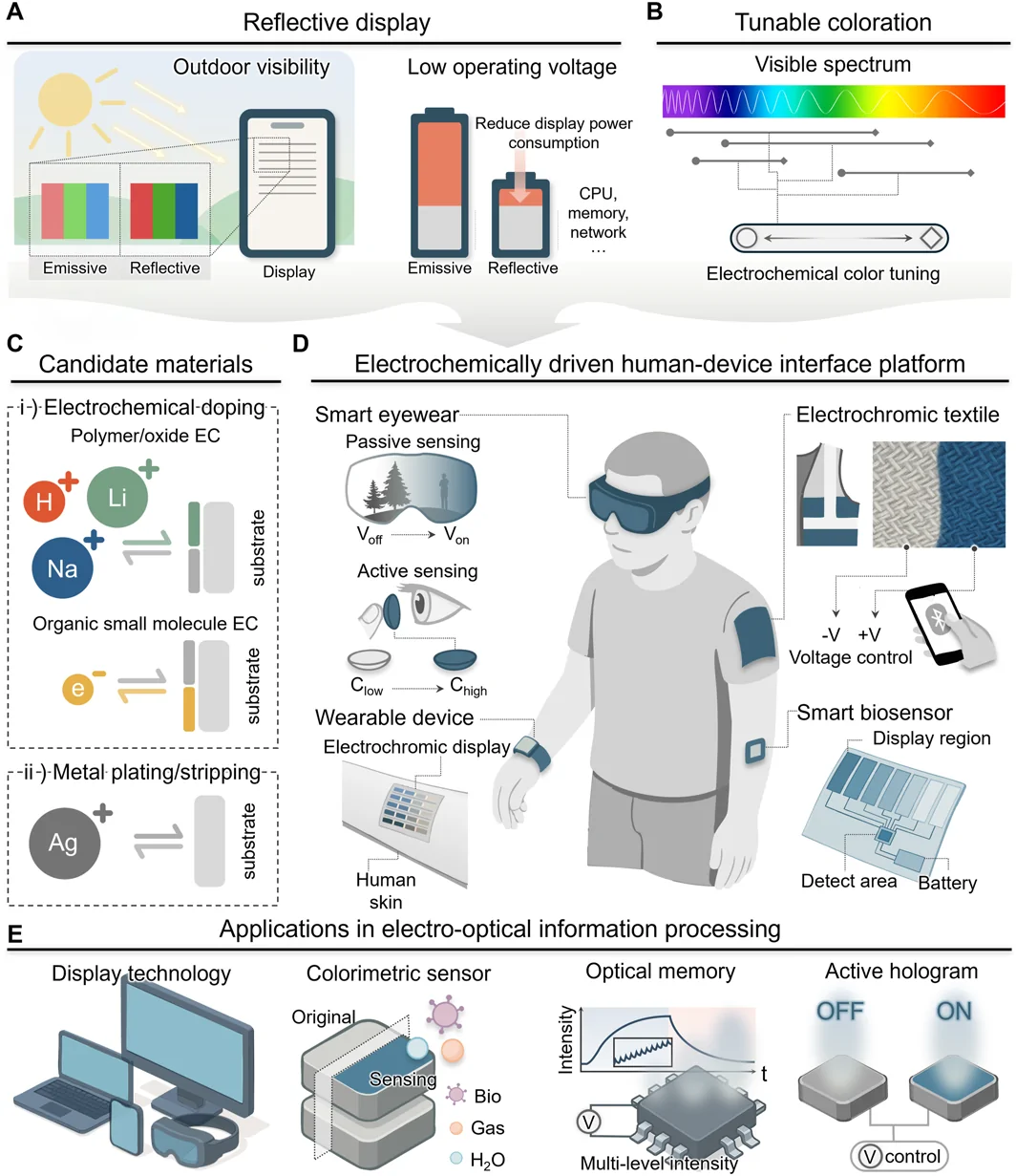
Overview of electrochemical approaches and strategies of tunable photonics. (A) Schematic illustration showing the advantages of reflective displays, including high visibility under bright ambient light and low‐voltage, energy‐efficient operation. (B) Illustration of tunable coloration enabled by electrochemically driven active materials, which modulate the visible spectrum in response to electrochemical stimuli. (C) Classification of electrochemically switchable material systems: (i) electrochemical doping materials and (ii) metal plating/stripping‐based systems. (D) Categorized schematics of human–device interface platforms utilizing electrochromic materials, including electrochromic textiles, smart eyewear, wearable devices, and smart biosensors. (E) Classification of electrochromic devices based on their key electro‐optical properties.

As shown in Figure 1C, we classify these materials into three categories according to their fundamental operating mechanisms: (i) materials based on electrochemical doping, including oxide‐ and polymer‐based EC materials as well as organic small‐molecule EC materials, in which charge transfer‐driven redox reactions modulate the optical parameters; and (ii) metals that utilize reversible plating/stripping to enable dynamic tuning of plasmonic resonances and the generation of structural coloration. When integrated into various photonic structures, these materials can be applied to a wide range of platforms, including smart eyewear [22, 23, 24], wearable devices [25], electrochromic textiles [26, 27], and biosensors [28, 29] (Figure 1D). Their dynamic tunability offers broad potential for display technologies [11, 25, 30, 31, 32, 33], low‐power colorimetric sensing [28, 29, 34, 35, 36], optical memory [37], and active holography [15, 17, 38, 39, 40] in next‐generation photonic and optoelectronic systems (Figure 1E).

## 2 Material and Structure Platform for Electrochemically Tunable Photonic Modulation

### 2.1 EC Material Candidates for Electrochemically Driven Reflective Displays

Electrochromic devices are generally composed of an EC layer, a working electrode (WE), a counter electrode (CE) that typically serves as a secondary electrode, and an electrolyte layer comprising an ion storage layer and/or an ion‐conducting layer (Figure 2A). Depending on the intended application, the electrodes can be engineered for mechanical flexibility, whereas the electrolyte layer can be formulated in solid, liquid, or gel states. Furthermore, reflective‐type EC devices incorporate a reflective layer composed of materials with high visible reflectance, ensuring durability and corrosion resistance under direct electrolyte exposure. The transparent ion storage layer and electrolyte facilitate rapid electrochemical modulation of the active material owing to their high ionic conductivity and well‐matched electrochemical potential windows. EC materials have attracted significant attention due to their low operating voltages and broad applicability compared with other active materials such as phase change material (PCM) [48, 49] and liquid crystal (LC) [7, 50, 51]. These materials are generally classified as either organic or inorganic materials and can also be categorized according to their chemical and optical modulation mechanisms induced: (i) electrochemical doping and (ii) metal plating/stripping.

**FIGURE 2:**
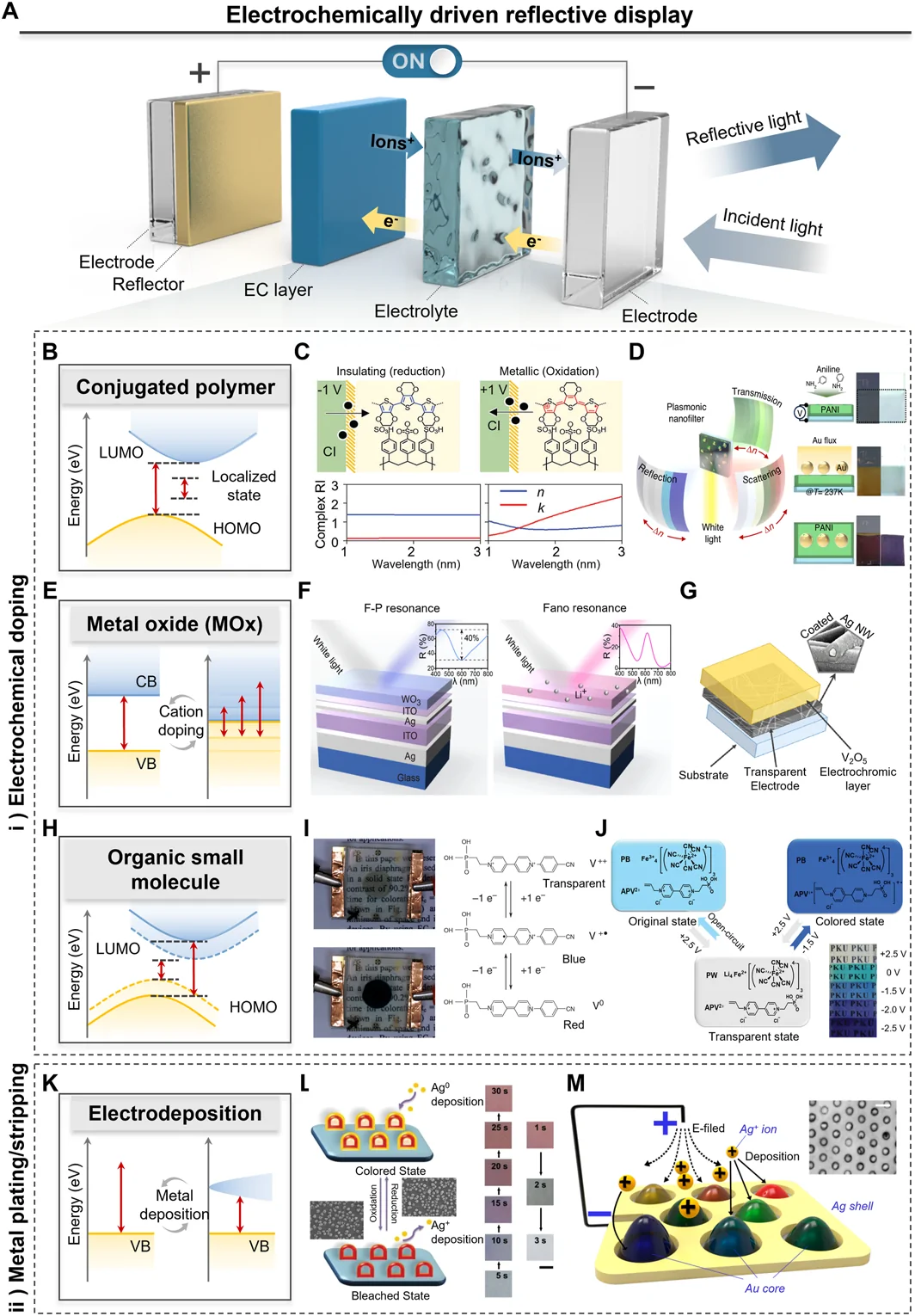
Electrochromic material candidates for electrochemically driven reflective displays. (A) Schematic of a traditional electrochemically driven reflective display. Ion or electron exchange occurs between the WE and CE under an applied voltage, enabling electrochemical doping or metal plating/stripping. The reflective layer returns ambient light, whereas the EC layer modulates the reflected spectrum through wavelength‐selective absorption. EC materials can be categorized into electrochemical doping‐based modulators (e.g., conjugated polymers, metal oxides, and organic small molecules) and ion/metal‐exchange systems (e.g., reversible plating/stripping metals). (B) Schematic of an energy‐level diagram of a conjugated polymer under external voltage bias. (C) Voltage‐dependent structural changes and modulation of the complex refractive index in PEDOT:PSS at −1 V and +1 V. Reproduced with permission [37]. Copyright 2024, Wiley‐VCH GmbH. (D) Fabrication sequence of a polyaniline (PANI)‐based Gires–Tournois (GT) resonator coupled with metal nanoparticles and the resulting color variation. Reproduced with permission [41]. Copyright 2024, Springer Nature Limited. (E) Illustrated schematic of energy‐level shifts of metal oxides (MO_x_) induced by cation doping. (F) Li^+^‐ions intercalation in WO_3_ and the corresponding change in the reflection spectrum. Reproduced with permission [42]. Copyright 2022, John Wiley & Sons Inc. or related companies. (G) Resonant cavity formed by an electrochromic V_2_O_5_ layer integrated with Ag nanowires. Reproduced with permission [43]. Copyright 2024, American Chemical Society. (H) Schematic energy diagram showing redox‐induced modulation of the HOMO–LUMO electronic structure in an organic small molecule. (I) Molecular structure of viologen illustrating its distinct radical cation state formed through electron‐driven redox switching, together with the associated coloration. Reproduced with permission [44]. Copyright 2025, Springer Nature Limited. (J) Viologen‐based electrochromic device integrated with Prussian blue and its corresponding color transitions. Reproduced with permission [45]. Copyright 2023, John Wiley & Sons Inc. or related companies. (K) Energy‐level evolution of metal ions enabling reversible metal plating/stripping under applied voltage. (L) Electrochromic device utilizing nanoparticles and Ag^+^ ions for dynamic color modulation. Reproduced with permission [46]. Copyright 2019, John Wiley & Sons Inc. or related companies. (M) Visible‐range optical modulation achieved by electric‐field‐driven Ag deposition onto Au‐core nanodomes. Reproduced with permission [47]. Copyright 2016, American Chemical Society.

Conjugated polymers, as representative organic electrochromic materials, possess several advantages, including mechanical flexibility, fast switching, low cost, and facile processability [52]. In the neutral state, conjugated polymers possess delocalized electrons occupying the highest occupied molecular orbital (HOMO) and lowest unoccupied molecular orbital (LUMO) levels, where π–π electronic transitions determine light absorption in the visible region (Figure 2B). Upon electrochemical doping, the electronic states are restructured, leading to a reduced HOMO–LUMO gap and a corresponding red shift in the absorption spectrum. Ko et al. designed an electrically controllable Tamm plasmon (ECTP) structure based on PEDOT:PSS, achieving efficient light absorption at targeted wavelengths (Figure 2C) [37]. The ECTP device exhibited theoretical absorption of up to 99% across the NIR range (800–2500 nm), with experimental absorption values of 88% at 1500 nm and 80% at 970 nm. The system successfully demonstrated 2‐bit optical memory functionality and neuromorphic applications. Furthermore, Kim et al. proposed a multilayer active plasmonic architecture by embedding Au nanoparticles within a conductive polyaniline (PANI) nanofilm (Figure 2D) [41]. The device exhibited electrochromic modulation across scattering, transmission, and reflection modes, producing distinct dichroic colors in both reflected and transmitted light. Furthermore, it achieved broadband color tuning across the visible spectrum under an applied voltage below 1 V with a switching time of 3.5 s, demonstrating its strong potential as a high‐efficiency electrochromic color filter.

Metal oxides (MO_x_), as representative inorganic electrochromic materials, exhibit intrinsic advantages of bistability and high optical contrast within the visible spectrum [53]. Upon electrochemical doping, ion intercalation into the lattice structure of transition metal oxides (TMOs) induces substantial spectral modulation through changes in the valence band (Figure 2E). Owing to their remarkable thermal and optical stability during repeated redox cycling, these materials are highly favorable for structural color generation and photonic device engineering. Tungsten oxide (WO_3_) is a representative inorganic electrochromic material that exhibits a reversible color transition from colorless to blue upon cathodic reduction [17, 54]. Chen et al. introduced a reconfigurable photonic architecture based on a hybrid resonator system that exploits the reversible electrochemical switching of WO_3_ (Figure 2F) [42]. The optical constants of WO_3_ can be dynamically modulated through Li^+^ intercalation, thereby enabling active control over the resonant characteristics of the structure. In its pristine state, the WO_3_‐based dual resonator exhibits Fabry–Pérot (F.–P.) resonance with distinct reflection peaks and transmission dips. Upon Li^+^ ion insertion, the device transitions to a Fano resonance, manifesting an asymmetric reflection profile indicative of interference between discrete and continuum modes. This electrically driven modulation of resonance characteristics underscores the potential of such systems for tunable trans‐reflective displays, adaptive optical filters, and active photonic modulators. In contrast, vanadium pentoxide (V_2_O_5_) is classified as an anodic electrochromic material, showing color transitions from yellow, green, and blue to brown, induced by progressively intercalating lithium ions into the lattice. Khan et al. demonstrated electrochromic functionality by sputter‐depositing V_2_O_5_ onto ZnO‐ and SnO_2_‐coated Ag nanowires (AgNWs), replacing conventional ITO transparent electrodes (Figure 2G) [43]. In particular, a V_2_O_5_ film deposited on a 30‐nm SnO_2_‐AgNW composite exhibited a distinct and stable color transition from orange to blue, maintaining over 500 switching cycles.

Viologens and their derivatives, as representative organic small molecules, possess superior structural flexibility and solution processability compared to their inorganic counterparts [55, 56, 57]. Upon redox reactions driven by electron injection, these materials form radical cation species that induce distinct color transitions among the dication (V^2+^), radical cation (V^1+^), and neutral state (V^0^), whereas molecular tailoring through functionalization or substitution enables systematic enhancement of color contrast and charge storage performance (Figure 2H). For example, Kortz et al. developed a high‐contrast electrochromic device by anchoring viologen and chemically complementary organic electrochromic molecules onto mesoporous electrodes via chemisorption [44] (Figure 2I). The device exhibited a large optical contrast (ΔOD ∼ 2.04 at 605 nm) and a fast‐switching speed of approximately 0.5 s, enabling a clear transition from a transparent to a black state. Additionally, it demonstrated excellent cycling stability, retaining over 83% of its initial contrast after 500 switching cycles. Moreover, Deng et al. developed a novel viologen compound, 1‐allyl‐1′‐(2‐phosphonoethyl)‐viologen (APV), by integrating a porous TiO_2_ film and an ion storage layer of Prussian blue (Figure 2J) [45]. The APV device demonstrated superior electro‐optical performance arising from the synergistic interaction between the viologen with the TiO_2_ layer and the Prussian blue film, achieving rapid switching times of 0.53/0.16 s and a high optical contrast (ΔT ∼ 77.5%). Moreover, the device exhibited a long‐term optical memory exceeding 24 h and remarkable cycling durability over 996 cycles, underscoring its strong potential for practical and durable electrochromic applications.

Metals inherently exhibit high refractive indices across the ultraviolet, visible, and infrared spectral regions. The collective oscillation of free electrons with incident electromagnetic waves generates plasmonic resonances (Figure 2K). At metal–dielectric interfaces, these interactions produce surface plasmon polaritons (SPPs), whereas confinement within subwavelength regions gives rise to localized surface plasmon resonances (LSPRs) with distinctive optical behavior. Reversible metal deposition enables dynamic tuning of these plasmonic modes, enhancing absorption at specific wavelengths and enabling optical modulation [58, 59]. Li et al. developed an electrically tunable electrochromic film capable of reversible transition between the colored and bleached states by controlling the redox behavior of Ag^+^/Ag (Figure 2L) [46]. The designed Au/Ag alloy shell served as a stable seed layer for selective Ag deposition, effectively suppressing random self‐nucleation of Ag nanoparticles. This alloy nanostructure significantly enhanced plasmonic resonance within the visible spectrum, resulting in high optical contrast and improved coloration performance. In addition, Wang et al. developed an electrochemically tunable Au/Ag core–shell nanodome array that enables real time full color plasmonic display operation through structural reconfigurability driven by reversible plating/stripping (Figure 2M) [47]. The plasmonic cell utilizes the reversible plating/stripping of Ag^+^ ions to realize dynamic control over the visible spectrum. In addition, the reconfigurable two‐electrode architecture facilitates a mechanically adaptive, chameleon‐inspired camouflage mechanism. This work broadens the application scope of electrochromic materials toward dynamically controllable photonic and display technologies.

### 2.2 Photonic Architectures Incorporating EC Materials for Optical Modulation

Various photonic architectures, including F.–P. [60, 61, 62], Bragg [63, 64], and Fano resonators [42, 65], have been extensively investigated in combination with electrochromic materials to enhance light–matter interactions. Such structural configurations effectively broaden the optical modulation bandwidth and enable precise control over device functionality, enabling progress toward next‐generation photonic applications [66]. In this review, these representative photonic structures are categorized into four types according to their fabrication complexity (Figure 3A). The degree of fabrication complexity plays a critical role in determining both the feasibility of large‐area implementation and the attainable pixel‐scale resolution.

**FIGURE 3:**
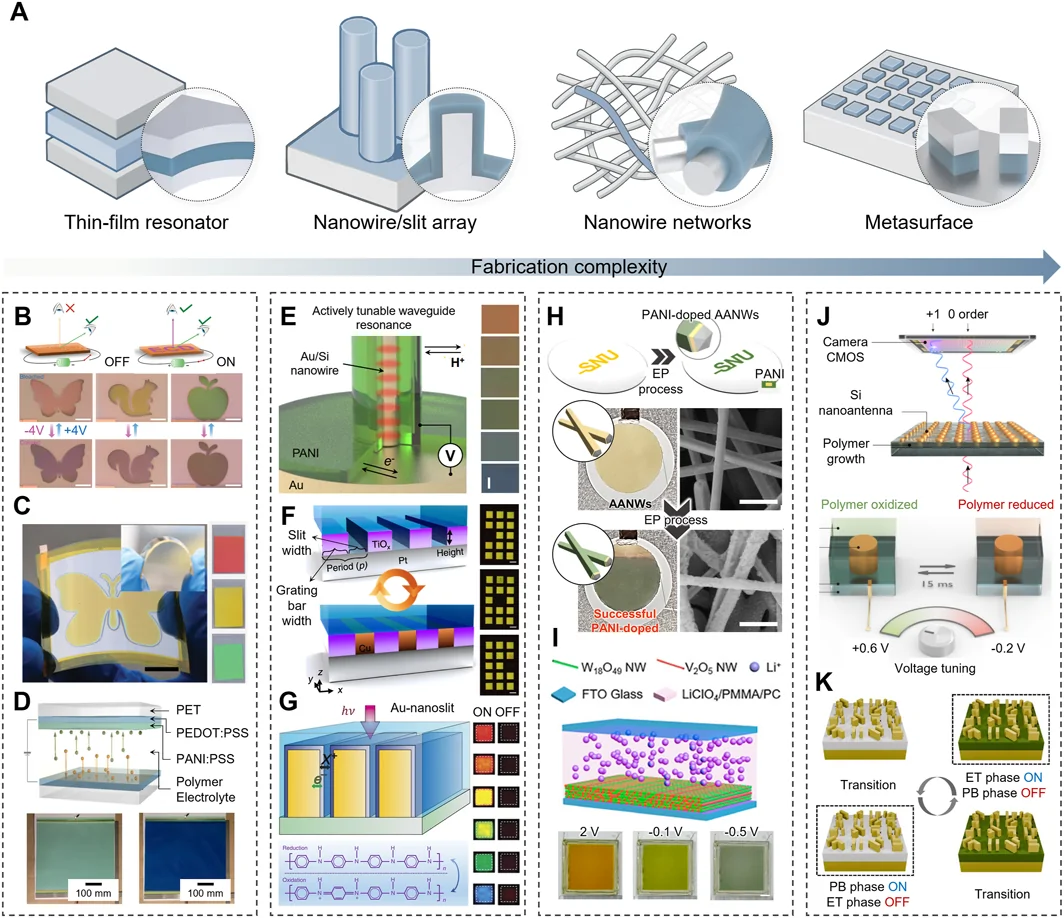
Photonic architectures incorporating electrochromic materials for optical modulation. (A) Illustrations of electrochromic‐integrated photonic architectures with increasing structural complexity, including thin‐film resonators, nanowire slit arrays, nanowire network structures, and metasurface platforms. (B) Angle‐ and voltage‐dependent visible‐light modulation achieved using a WO_3_‐based thin‐film resonator structure. Reproduced with permission [10]. Copyright 2024, John Wiley & Sons Inc. or related companies. (C) High‐optical‐modulation, high‐purity‐color WO_3_‐based flexible electrochromic device. Reproduced with permission [30]. Copyright 2020, Springer Nature Limited. (D) Electrochromic device integrating anodic and cathodic materials for complementary color tuning. Reproduced with permission [67]. Copyright 2024, Springer Nature Limited. (E) Dual‐mode resonant device combining a nanowire array with PANI, enabling electrically switchable colors. Reproduced with permission [11]. Copyright 2023, De Gruyter Brill. (F) Addressable device utilizing slit structures and reversible metal‐ion deposition. Reproduced with permission [12]. Copyright 2024, Springer Nature Limited. (G) Full‐color optical device based on Au nanoslit structures integrated with conductive polymers. Reproduced with permission [13]. Copyright 2016, Springer Nature Limited. (H) PANI‐based core–shell nanowires capable of modulating absorption across the visible–NIR spectrum. Reproduced with permission [68]. Copyright 2023, Royal Society of Chemistry. (I) High‐visibility device employing a nanowire film composed of W_18_O_49_ and V_2_O_5_. Reproduced with permission [69]. Copyright 2021, American Chemical Society. (J) Low‐power holographic device using electrochromic PANI. Reproduced with permission [14]. Copyright 2024, De Gruyter Brill. (K) Optical encryption device with two independent tunable parameters. Reproduced with permission [15]. Copyright 2025, John Wiley & Sons Inc. or related companies.

The most fundamental configuration, the F.–P. resonator, features a planar thin‐film structure in which the electrochromic layer is stacked between bottom and top metal layers. This architecture is suited for large‐area colorimetric applications, and its active layer has been demonstrated to integrate well with flexible substrates and gel electrolytes, enabling the development of mechanically flexible systems. For example, Zhang et al. designed an all‐solid‐state electrochromic device based on WO_3_, demonstrating an expanded range of structural color tunability (Figure 3B) [10]. In their design, CeO_2_ was employed as the ion storage layer and LiNbO_3_ as the solid electrolyte, ensuring efficient ion exchange and improved practicality compared with conventional aqueous electrolyte systems. By precisely controlling the thicknesses of three dielectric layers, the device achieved multiple distinct color states. Owing to the inherent stability of inorganic electrochromic materials, the device maintained 84% of its modulation contrast after 7200 cycles, confirming excellent durability and optical memory behavior. Furthermore, by exploiting the angle‐dependent characteristics of the optical structure, the device exhibited camouflage and encryption functionality at +4 V, whereas at −4 V, enhanced contrast patterns were achieved for readability and display performance. Wang et al. demonstrated a broadened color gamut by varying the reflective layer material and precisely tuning the thickness of the WO_3_ layer to achieve diverse reflected colors (Figure 3C) [30]. Under applied voltages ranging from −0.8 to 0.5 V, the device exhibited a pronounced reflectance peak shift from 760 to 517 nm (243 nm), confirming a wide optical modulation range. Moreover, the reflected color remained nearly unchanged across incident angles from 0° to 40°, indicating excellent angular color stability. These results highlight a structurally simple yet highly tunable system, underscoring its strong potential for practical electrochromic display applications. In addition, EC materials exhibit excellent modulation capability not only in the visible range but also in the near‐infrared (NIR) region. For example, Park et al. developed a low‐power smart window that integrates anodic (PANI:PSS) and cathodic (PEDOT:PSS) electrochromic materials within a single device, achieving high optical contrast and efficient modulation through complementary electrochromic behavior (ΔT ∼ 52% at 600 nm) (Figure 3D) [67]. By integrating the structure onto a flexible substrate and utilizing a roll‐to‐roll fabrication process, large‐area production (500 mm × 500 mm) was successfully achieved. Under infrared (IR) illumination, the PEDOT:PSS‐ and PANI:PSS‐based EC window showed only a 3.5°C increase under identical conditions, demonstrating effective near‐infrared (NIR) absorption and improved thermal regulation.

In contrast, micro/nano‐3D photonic structures coupled with surface‐deposited electrochromic polymers exhibit enhanced optical responses through surface plasmon polariton (SPP) coupling. Ko et al. fabricated an electrochromic device by electrodepositing PANI onto Si nanowires with a reflective layer, achieving a broad optical modulation range spanning from red to blue tones (Figure 3E) [11]. Depending on the oxidation state of the surface‐deposited PANI layer, two resonance modes were identified: an SPP mode that guides incident light along the surface and an ultrathin resonator mode, which produced vivid and well‐defined structural colors. By tuning the spacing between nanowires, various structural colors were realized, and the nanowire array was further demonstrated as a unit cell for optical information encryption. Moon et al. demonstrated a maximum optical contrast of 97% by integrating a TiO_2_ grating deposited on a Pt substrate with the reversible plating/stripping of Cu^+^ ions (Figure 3F) [12]. The optical response arises from the interaction between the waveguide‐array mode supported by the high‐contrast grating and the s‐polarized mode activated during Cu^+^ insertion. The s‐polarized mode produces a sharp spectral peak, yielding highly saturated structural colors. The device maintained stable color switching over 540 cycles and exhibited addressable operation in a 3 × 5 pixel array with vivid and high‐contrast coloration. Xu et al. demonstrated monochromatic and full‐color electrochromic operation with high contrast and fast switching by integrating periodic metal nanoslit arrays (Au and Al) with electrochromic polymers (PANI and PolyProdot‐Me_2_) (Figure 3G) [13]. The Au nanoslit combined with PANI exhibited clear on/off switching within an applied voltage range of −0.2 to 0.3 V. Furthermore, by tuning the period of the Al nanoslit array, color pixels spanning red to blue were displayed. Using the high‐coloration‐efficiency polymer PolyProdot‐Me_2_, uniform optical attenuation was achieved across individual pixels. The device showed a fast‐switching speed (∼80 ms) and an optical contrast ranging from 73% to 90% across the visible spectrum.

Metal nanowire networks provide continuous conductive pathways, facilitating faster electron transport and ion exchange. Jung et al. achieved stable and reversible electrochromism by conformally coating PANI onto Au nanowires, forming a core–shell nanowire architecture that maintained its mechanical integrity even after over 1000 bending cycles (Figure 3H) [68]. Integration of this structure with printed circuit board (PCB) technology enabled the fabrication of a multipixel display, demonstrating its potential for security display applications operating in both the visible (Vis) and infrared (IR) spectral regions. Moreover, the low‐energy thin‐film configuration exhibited wide optical modulation in the Vis–IR range. Wang et al. developed an electrochromic display based on a nanowire film composed of W_18_O_49_ and V_2_O_5_, achieving high optical transmittance and vivid color contrast (Figure 3I) [69]. By adjusting the composition ratio of the two electrochromic nanowires, the color gamut was significantly expanded. The device further demonstrated its potential for display applications by individually addressing patterned regions, enabling enhanced visibility and color differentiation.

Metasurfaces with active materials have recently emerged as promising low‐power, multifunctional photonic platforms capable of encoding color information at various pixel scales and enabling high‐density integration for advanced optical and display applications. Lu et al. demonstrated efficient optical modulation ranging from 10% to 90% within a low operating voltage window of −0.2 to 0.6 V (Figure 3J) [14]. The device enabled practical implementations of dynamic holography and beam steering and maintained stable operation over 2000 switching cycles, confirming its robustness and applicability for reconfigurable photonic systems. Kim et al. developed a low‐power holographic encryption device based on a dual‐channel metasurface governed by an electroactive chiral optical response (EACOR) (Figure 3K) [15]. By employing two independent control parameters, the polarization channel (LCP or RCP) and the voltage channel (−0.2 V or 0.3 V), the system exhibited four discrete holographic states corresponding to different oxidation levels of PANI. The incorporation of a conductive polymer into the metasurface framework demonstrates its potential for electrically reconfigurable optical encryption and multifunctional photonic display technologies.

As shown in Table 1, we have summarized electrochemically tunable materials and their corresponding driving mechanisms. Although the table does not include all possible material and photonic structure combinations, several representative materials can be applied to a wide range of photonic architectures and used to realize additional optical functionalities. In this review, plasmonic arrays include both nanowire/slit arrays and nanowire network structures. By selecting appropriate modulation parameters in combination with different optical materials and photonic structures, these systems can be adapted for specific application requirements.

**TABLE 1:** Overview of electrochemically tunable materials and photonic structures for optical modulation.

Electrochemical mechanism	Material	Photonic structure	Modulated optical property	Representative application	Ref.
Ion insertion	WO_3_	Fabry–Pérot cavity	Reflectance/color	Reflective display	[30]
	WO_3_	Metasurface	Reflectance/color	Reflective display	[32]
	V_2_O_5_	Plasmonic array	Reflectance/color	Reflective display	[43]
Redox reaction	PANI	Fabry–Pérot cavity	Reflectance/color	Reflective display	[33]
	PEDOT:PSS	Plasmonic array	Resonance wavelength	Synaptic device/IR display	[37]
	PEDOT:PSS	Metasurface	Phase/polarization	Beam steering/holography	[38]
Metal plating/stripping	Cu	Plasmonic array	Reflectance/color	Reflective display	[12]
	Ag	Plasmonic array	Reflectance/color	Reflective display	[46]
	Ag	Plasmonic array	Reflectance/color	Reflective display	[47]

## 3 Application of Electrochemically Driven Tunable Photonics

### 3.1 Electrochemical Light Modulations

Electrochemical optical modulation exhibits scale dependent precision and functional tunability, with the degree of control defining the achievable color uniformity and application scope [70]. The modulation strategies can be systematically organized by control scale into single unit operation, pixel arrays, and large‐area system‐level integration. At the most fundamental level, color modulation is induced through changes in the complex refractive index (*n*) or via carrier injection within an individual electrochemical cell (Figure 4A) [71, 72, 73]. This localized tuning mechanism facilitates a broad color gamut with high spectral purity. Notably, inorganic electrochromic materials that undergo refractive index modulation through ion intercalation provide a fundamental platform for single unit electrochemical color control. Wang et al. demonstrated an ultracompact, asymmetric F.–P. nanocavity electrochromic device utilizing partially reflective tungsten (W) as both the current collector and mirror (Figure 4B, top) [30, 74]. The optical system consists of a thin WO_3_ layer sandwiched between reflective and semitransparent interfaces, where strong optical interference is induced through the controlled *n*, *k* variations of WO_3_ upon ion insertion. This nanoscale interference modulation enabled vivid, structurally defined colors—including blue, red, and yellow—without external filters or pigments (Figure 4B, bottom). This interference‐enhanced architecture enables single unit electrochemical modulation of the optical constants within a minimal device geometry and directly links material‐level ion dynamics to macroscopic color expression. Coupling plasmonic resonators with electroactive media enables electrochemical programmability of the intense light–matter interaction in nanoscale gaps, thereby delivering single nanopixel color control while preserving scalability. Peng et al. integrated an electrically tunable active medium into nanoscale plasmonic gaps to realize electrically driven plasmonic metasurfaces, achieving precise color modulation at the single nanoparticle (NP) (Figure 4C, top) [31, 52]. A core–shell structure (PANI with Au NP) coupled to a gold mirror supports hybridized gap‐plasmon resonances at hot spot regions that are dynamically tuned by the applied potential. Upon oxidation of PANI, the scattering spectrum exhibits a red shift exceeding 100 nm. The platform exhibits polarization‐ and angle‐insensitive operation, featuring high optical contrast (> 50%), fast modulation speed (> 50 Hz), and bistable color states that are sustained for several hundred seconds. Combining vivid color retention with ultralow‐energy operation, this work verifies the scalability of electrochemical color modulation from individual nanopixels to wafer‐scale metasurfaces (Figure 4C, bottom). More broadly, when subwavelength resonators are co‐integrated with waveguide and cavity modes, redox‐active media permit intermodal switching among distinct resonances, yielding low‐voltage, multifunctional color control and high‐capacity encoding. Ko et al. developed electrochromic nanopixels based on PANI‐integrated hybrid nanowires (Figure 4D, top) [11]. Each nanopixel supports two distinct resonances—a surface plasmon polariton (SPP)‐assisted waveguide resonance that acts as a wavelength‐selective absorber and an ultrathin cavity that provides broadband absorption. By electrochemically switching between these states, vivid color transitions from red to green and blue were achieved at sub‐1 V, compatible with CMOS circuitry (Figure 4D, bottom). The color modulation arises from the redox‐driven optical transition of PANI, shifting its optical constants from lossy to dielectric, thereby strengthening light–matter interaction within the hybrid nanostructure. Fabrication was scaled to an 8‐inch wafer, enabling multibit optical encryption (up to 8 bits per unit) and further extending to 10 bits per nanopixel via angle‐multiplexed spectral encoding. The study highlights an energy‐efficient, dual‐mode electrochromic architecture capable of precise and multiplexed color modulation within subwavelength thickness devices.

**FIGURE 4:**
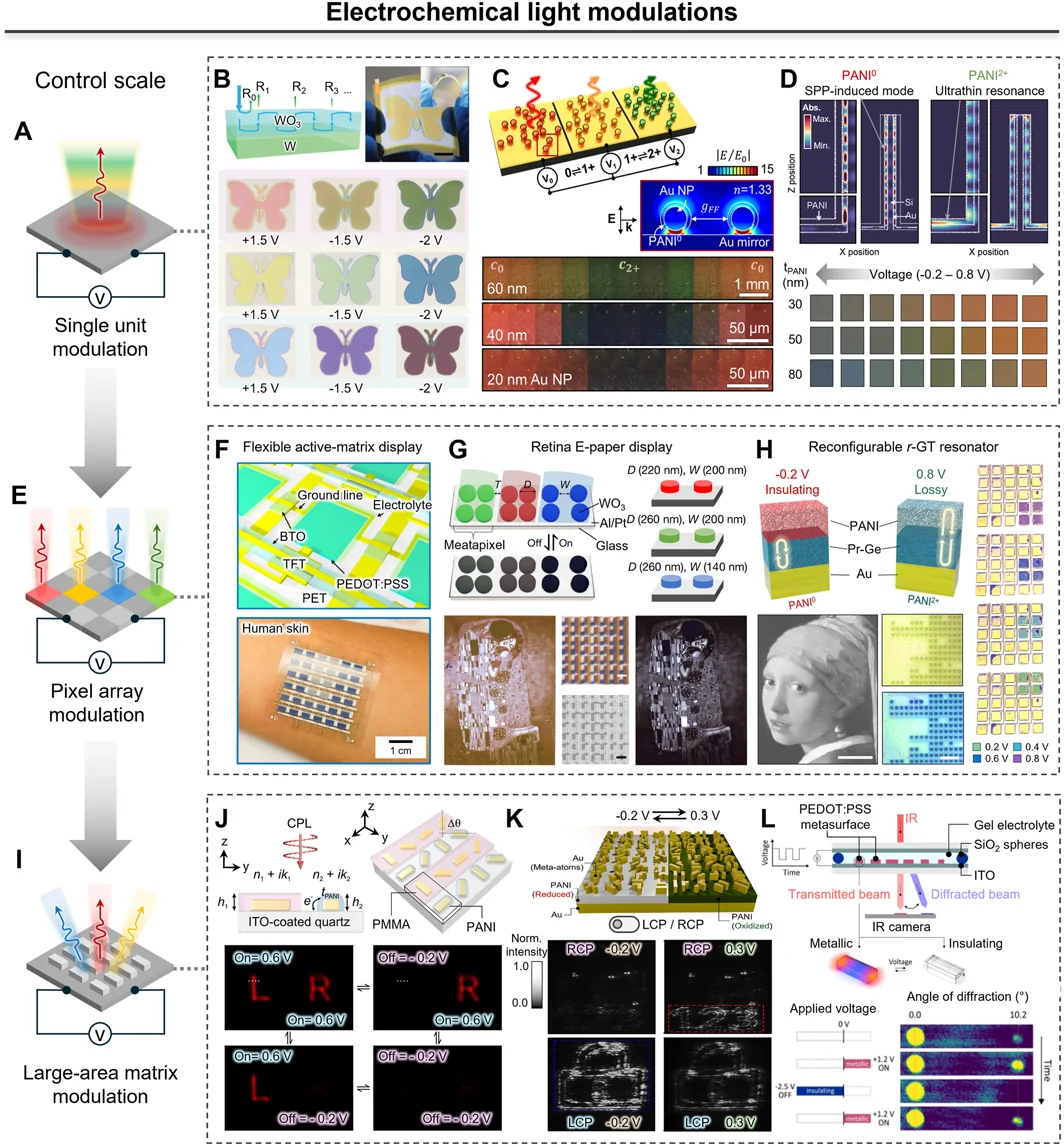
Electrochemically programmable color/phase control across scales. (A) Schematic illustration of single unit control electrochemical optical modulation. (B) Ultracompact asymmetric F.–P. nanocavity (W/WO_3_) showing ion insertion‐driven interference colors (top) and the resulting structural color changes (bottom). Reproduced with permission [30]. Copyright 2020, Springer Nature. (C) Structure of an electrically driven plasmonic metasurface based on PANI with Au nanoparticle (top) and the gap‐plasmon‐mediated color modulation produced by electrochemical tuning (bottom). Reproduced with permission [31]. Copyright 2019, American Association for the Advancement of Science. (D) Voltage‐driven mode switching of hybrid nanowire electrochemical nanopixels (top), with the resulting bias‐dependent structural colors (bottom). Reproduced with permission [11]. Copyright 2023, De Gruyter Brill. (E) Pixel arrays enabling spatially addressable electrochromic modulation. (F) Schematic of a screen‐printed active‐matrix EC display with CNT‐TFT backplane and PEDOT:PSS pixels (top) and the fabricated panel under operation (bottom). Reproduced with permission [25]. Copyright 2016, American Chemical Society. (G) WO_3_ nanodisk metapixels unit‐cell schematic with tunable thickness (*T*), disk diameter (*D*), and wall width (*W*) (top). Artwork rendering produced with the metapixel palette, with a magnified mosaic region and a reference tile (bottom). The scale bar is 2 μm. Reproduced with permission [32]. Copyright 2025, Springer Nature. (H) PANI‐integrated resonant monopixel based on Au/P_r_‐Ge/PANI structure (left, top). Artwork rendering and pixel‐matrix demonstrations with rewriteable multilevel operation (left, bottom and right). The scale bar is 15 μm. Reproduced with permission [33]. Copyright 2025, Springer Nature. (I) Architecture illustrating scaling to wafer‐scale, system‐level integration. (J) Electroactive chiral metasurface with PANI‐coated Au unit cells on ITO/quartz (top). Polarization‐resolved hologram confirms rewritable operation with clear contrast (bottom). Reproduced with permission [17]. Copyright 2021, American Association for the Advancement of Science. (K) PANI‐integrated Au metasurface enabling bias‐controlled index tuning and LCP/RCP selective imaging (top) and switching from −0.2 to 0.3 V modulates the chiral response, revealing distinct features in each polarization channel (bottom). Reproduced with permission [15]. Copyright 2025, Wiley‐VCH GmbH. (L) PEDOT:PSS metasurface with a gel‐polymer electrolyte and ITO electrodes for solid‐state tuning (top). Square‐wave bias switches states and steers IR diffraction from 0° to ∼10° (bottom). Reproduced with permission [38]. Copyright 2023, De Gruyter Brill.

Extending from single unit control to array‐level operation, spatially addressable color modulation is realized using microelectrode pixel matrices (Figure 4E). Individual pixel addressing enables multicolor imaging and programmable pattern displays [75, 76, 77]. Robust array performance hinges on three factors: electrode architecture, pixel‐level electrolyte isolation/compartmentalization to suppress cross talk, and uniform potential distribution—requirements that motivate high‐precision lithography and strict materials compatibility [78, 79]. Cao et al. reported a fully screen‐printed active‐matrix electrochromic display based on carbon‐nanotube (CNT) thin‐film transistors (TFTs) [25, 80]. Each pixel integrates a PEDOT:PSS electrochromic layer with a printed electrolyte to realize ion‐insertion/extraction‐driven color modulation (Figure 4F, top). Monolithic integration of the printed CNT‐TFT backplane with the electrochromic pixel array yields individually addressable pixels on large‐area, flexible substrates with stable, repeatable switching characteristics (Figure 4F, bottom). End‐to‐end printing affords low‐cost manufacturability, scalability, and long‐term stability, highlighting suitability for disposable tags, medical electronics, and smart‐home devices. This printed‐electronics pathway co‐optimizes materials, processes, and device architecture—uniform potential distribution, pixel‐level electrolyte isolation, and scalable fabrication—to meet the demands of pixel‐array electrochromic modulation. Santosa et al. extended these design tenets to the nanophotonic regime by realizing electrochromic metapixels that translate electrochemical index–absorption tuning into sub‐µm, diffraction‐limited pixels, thereby bridging single unit resonators and lithographic microarrays [12, 32, 81]. The architecture comprises WO_3_ nanodisks on a metallic reflector (Al/Pt) with a laterally compact WE‐CE geometry (∼500 nm gap) that shortens ionic pathways (Figure 4G, top). Reversible electrochemical reduction couples refractive‐index and absorption changes, yielding a ∼560‐nm metapixel pitch (> 25,000 ppi), high reflectance (∼80%), and ∼50% optical contrast under ambient illumination, whereas power is expended mainly during state updates (∼0.5–1.7 mW cm^−2^). This transport‐optimized layout enables video‐rate operation (> 25 Hz), reaching ≳ 95% of the contrast swing with ∼40 ms. WO_3_ metapixel palette‐based artwork rendering demonstrates retina‐level spatial density when the display aperture approaches the pupil diameter and full‐color dynamic playback, complementing the printed active‐matrix approach discussed above (Figure 4G, bottom). At the extreme end of spatial density with minimal routing overhead, a resonant monopixel approach delivers full‐color modulation with memory‐in‐pixel and simplified addressing. Ko et al. reported an electrically reconfigurable Gires–Tournois (*r*‐GT) resonator co‐integrated with an electroactive polymer, PANI (Figure 4H, top) [33]. The device enables complex refractive index modulation across the visible spectrum range within a sub‐1‐V (−0.2 to 0.8 V), producing vivid hue shifts beyond complementary ranges at a power density of ∼90 μW cm^−2^. The architecture is inherently scalable—from ultrahigh pixel densities (∼16,900 ppi) to wafer‐level fabrication—and supports passive‐matrix addressing, allowing individual pixel control without high‐voltage drivers. A high‐resolution artwork (Figure 4H, bottom) and programmed patterns in a 5 × 5 passive‐matrix panel (Figure 4H, right) were rendered while preserving memory‐in‐pixel retention; the measured electro‐optical response reached ∼34 ms rise (oxidation) and ∼171 ms fall (reduction), consistent with video‐rate content. Taken together, the *r*‐GT monopixel platform combines low‐voltage operation, broad spectral tunability, passive addressing, and scalable processing for dense, full‐color electrochromic displays.

At the final stage, the system can scale to a large‐area matrix architecture, where frame rate, energy efficiency, spatial uniformity over large‐areas, and wiring/interconnect density become dominant engineering constraints (Figure 4I) [82, 83]. Such platforms enable metasurface‐based structural color control and dynamic wavefront shaping, extending functionality beyond simple color modulation to multipixel visual information encoding and dynamic holography. As a representative example of scalability toward large‐area arrays, Kissner et al. locally grafted PANI onto preselected gold nanorods to realize electrochemically controlled metasurfaces operating at visible wavelengths, enabling in situ phase programming with high contrast, fast response, and excellent reversibility (Figure 4J, top) [17]. Within an aqueous electrochemical cell, subwavelength resonator unit cells undergo redox‐mediated PANI growth and cycling that modulate the phase of individual elements, thereby allowing bias‐controlled, wafer‐level reconfiguration of the metasurface phase profile (Figure 4J, bottom). This approach demonstrates anomalous transmission and dynamic holography as representative functions, establishing that unit‐cell‐level polymer redox can translate directly into array‐level wavefront engineering. Kim et al. integrated a thin PANI layer with chiral Au unit cells to realize an electroactive metasurface on a reflective metal‐insulator‐metal (MIM) platform, wherein the electroactive chiral optical response enables control of an exceptional topological phase under low‐voltage operation (Figure 4K, top) [15]. One control channel arises from the intrinsic, chirality‐dependent response of the unit cells, whereas the second exploits voltage‐induced refractive index modulation of PANI in an aqueous electrolyte, enabling electrical addressing. This dual‐channel scheme achieves reversible switching of ∼52 ms at 0.5 V and is validated through a dual‐channel hologram that demonstrates independent control of the polarization and voltage channels within a matrix‐compatible architecture for optical encryption and display applications (Figure 4K, bottom). Meanwhile, reliance on liquid electrolytes introduces practical challenges—particularly in packaging, pixel isolation, and matrix addressing. Aqueous electrolyte systems deliver high‐contrast, fast, and reversible operation, but their handling complicates integration. By contrast, solid‐like gel‐polymer electrolytes enable compact, self‐contained, CMOS‐compatible platforms that are better suited for scalable, large‐area arrays. De Jong et al. demonstrated an electrically switchable, miniaturized metasurface that integrates PEDOT:PSS with a gel‐polymer electrolyte, enabling reversible dielectric‐metallic switching under square‐wave bias (Figure 4L, top) [38]. The device electrically toggles plasmonic resonances in the 2–3‐μm band to achieve beam steering up to approximately 10° (Figure 4L, bottom) and supports switching frequencies up to 10 Hz with transients of ∼42 ms (oxidation) and ∼57 ms (reduction). This CMOS‐compatible, solid‐state implementation mitigates the practical limitations of liquid cells, establishing a pathway toward matrix‐addressable metasurfaces and, ultimately, submicrometer‐pixel spatial light modulators and reconfigurable holographic displays. These advances mark a transition from single unit material response control to coordinated, multidimensional optical information encoding, establishing electrochemical color and phase modulation as a scalable platform poised for high‐resolution displays, security patterning, colorimetric sensing, and optical memory.

### 3.2 Electrochemical Control of Nonvolatile Photonics

Next, we consider electrochemically driven optical memory and programmable photonics. Electrochemically active materials enable nonvolatile state storage, programmable phase/amplitude control, and multilevel optical operations by leveraging reversible compositional changes and tunable *n*, *k* values [84, 85, 86]. To be viable, such systems must satisfy stringent criteria—high on/off ratio for noise robustness, fast switching with low energy consumption, endurance under repeated computation/training, and long‐term state retention—as illustrated in Figure 5A [86]. In this section, we adopt these performance metrics as a common framework to compare demonstrations of optical memory (state retention and multilevel encoding) and programmable photonics (electrochemical reconfiguration of phase/amplitude maps) and to discuss co‐optimization strategies spanning materials, drive schemes, and device architecture.

**FIGURE 5:**
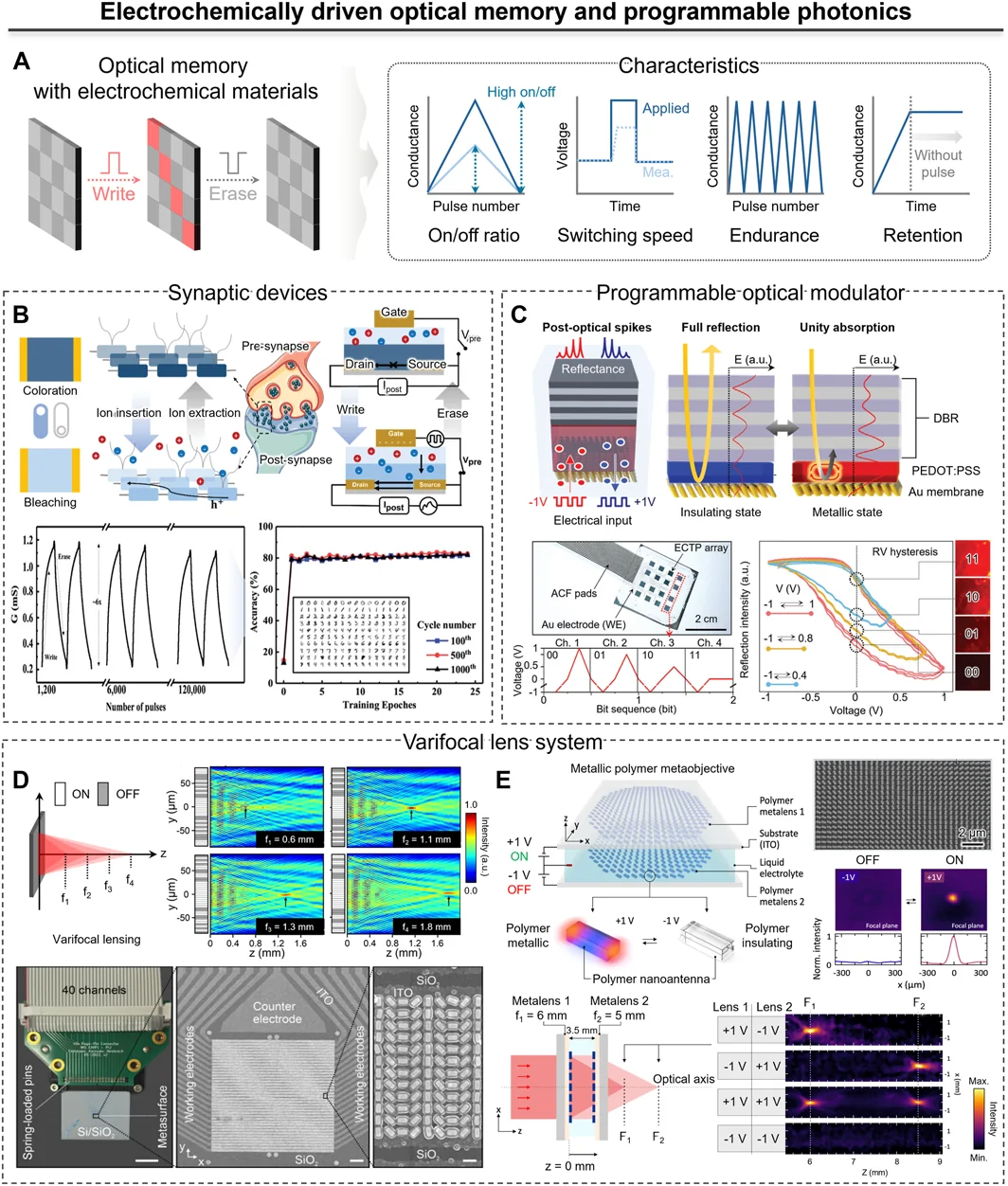
Electrochemical photonic memory and programmability. (A) Write/erase operation and key metrics of optical memory with electrochemical materials. (B) Ion‐driven synaptic elements enabling analog, nonvolatile conductance updates (top) and endurance cycling and low‐noise training accuracy plots (bottom). Reproduced with permission [87]. Copyright 2023, Wiley‐VCH GmbH. (C) PEDOT:PSS Tamm plasmon cavity switching between insulating and metallic states (top) and R–V hysteresis and deterministic 2‐bit array programming (bottom). Reproduced with permission [37]. Copyright 2024, Wiley‐VCH GmbH. (D) Electrochemical addressing of discrete focal states shown in axial intensity maps (top) and device micrographs of electrodes and electrolyte compartments (bottom). Reproduced with permission [39]. Copyright 2024, American Chemical Society. (E) Electrolyte‐gated polymer metasurface toggling plasmonic ON/insulating OFF (top) and voltage‐programmed metalenses with plane‐selective focusing and multiplexed imaging (bottom). Reproduced with permission [40]. Copyright 2022, Springer Nature.

Electrochemically driven optical memory spans multiple device classes, with ion‐programmable synaptic devices as a representative example. Leveraging reversible ion insertion/extraction and mixed ionic‐electronic conduction, these devices offer analog, nonvolatile state programming and have the potential to meet the key metrics for practical optical memories. Roh et al. bridged electrochromic polymers (ECPs) and neuromorphic memory by translating ECP redox physics into electrochemical RAM (ECRAM) operation (Figure 5B, top) [87]. Polymer–ion interactions, redox activity, mixed conduction, and stability are mapped to device‐level figures of merit, and the optimal ECP–electrolyte pair is implemented in a single transistor ECRAM. The device exhibits linear, symmetric conductance updates with a large dynamic range (∼1 mS, ∼6 ×), and low write noise, yielding > 84% training accuracy on a standard image‐recognition dataset (Figure 5B, bottom). Within the context of electrochemically driven optical memory and programmable photonics, this materials‐by‐design approach addresses long‐standing challenges of asymmetric weight updates and limited dynamic range while remaining compatible with photonic readout/integration pathways. As a complementary class to synaptic memory, we highlight programmable optical modulation enabled by electrochemical control of the complex refractive index. By reversibly tuning *n*, *k* in an adjacent electroactive layer under low bias, such modulators support deterministic programming with analog, nonvolatile updates and low energy cost—offering a direct pathway to array‐compatible, device‐level reconfiguration. Ko et al. reported a sub‐1‐V, electrically programmable Tamm plasmon modulator comprising a DBR‐Au cavity coupled to a PEDOT:PSS electroactive layer (Figure 5C, top) [37]. Electrochemical doping/de‐doping enables near‐IR, amplitude‐dominant modulation with > 88% reflectance contrast, approaching perfect absorption at the epsilon‐near‐zero condition. Array‐level programmability is demonstrated by channel‐selective voltage sweeps that deterministically write 2‐bit states (00/01/10/11), producing characteristic RV hysteresis and four well‐separated, 0‐V‐readable levels (Figure 5C, bottom). In our evaluation framework, this platform satisfies large contrast at CMOS‐compatible voltages, analog and nonvolatile state storage, and array‐compatible operation, positioning active Tamm plasmon modulators as a practical route to programmable photonics.

Device‐level amplitude programmability establishes the feasibility of electrochemical control at CMOS‐level voltages with analog, nonvolatile updates. Building on this, we next consider electrochemically programmable metasurfaces that provide independent per‐pixel control of the complex refractive index and therefore, direct write access to the optical phase map.

Elevating programmability from a single resonator to array‐scale wavefront synthesis enables varifocal lensing and scene‐adaptive imaging without moving parts, aligning naturally with our evaluation framework while introducing system‐level figures of merit (aperture, numerical aperture, focal‐tuning range, and wavefront error). Kassiner et al. demonstrated an electrochemically programmable metasurface with independently addressable pixels at optical frequencies (Figure 5D) [39]. PANI locally integrated on Au nanorods and wired to pixel electrodes undergoes reversible electrochemical doping/de‐doping, reprogramming the per‐pixel propagation phase (and associated amplitude) on demand. On the same chip, compiled phase maps realize dynamic beam steering and on/off‐axis varifocal lensing, evidencing array‐compatible wavefront control without moving parts. This work explicitly addresses the phase dimension of programmability while preserving pixel‐level addressability and device‐level reconfigurability. The metasurface enables per‐pixel electrochemical phase programming, whereas the metalenses‐on‐demand architecture integrates these programmable phase maps into a deployable, nonvolatile varifocal metaobjective—thereby linking device‐level control to system‐level imaging. Karst et al. presented electrically switchable metalenses on demand based on metallic‐polymer plasmonic nanoantennas (Figure 5E) [40]. A CMOS‐level (± 1 V) bias induces an optical metal‐to‐insulator transition in the polymer, reversibly toggling plasmonic resonances on/off and thereby writing lens phase profiles without mechanical moving parts. Two reconfigurable lenses are combined into an electroactive, nonvolatile, multifunctional metaobjective whose optical states can be set on demand, enabling mechanics‐free focal tuning and axial scanning. The platform exemplifies switchable metasurfaces that deliver high‐efficiency control on subwavelength scales, pointing to compact, low‐power building blocks for integrated augmented reality (AR)/virtual reality (VR)‐class metaoptics.

### 3.3 Electrochemically Driven Colorimetric Sensors

Electrochemical colorimetric platforms transduce environmental stimuli into visible color by modulating the optical states of an active layer integrated with a resonant or pixelated structure. Readout modalities include calibrated color‐difference metrics, controlled hue rotations, and addressable pixel patterns for hidden or spatially encoded displays. The approach offers practical advantages—near‐zero standby power with low‐energy updates, inherently human‐ and camera‐readable outputs, straightforward scaling to matrix arrays, and fast, reversible cycling (Figure 6A). Implementations are commonly organized by target analytes, encompassing gas detection, biosensing, and environmental ion monitoring [88, 89, 90].

**FIGURE 6:**
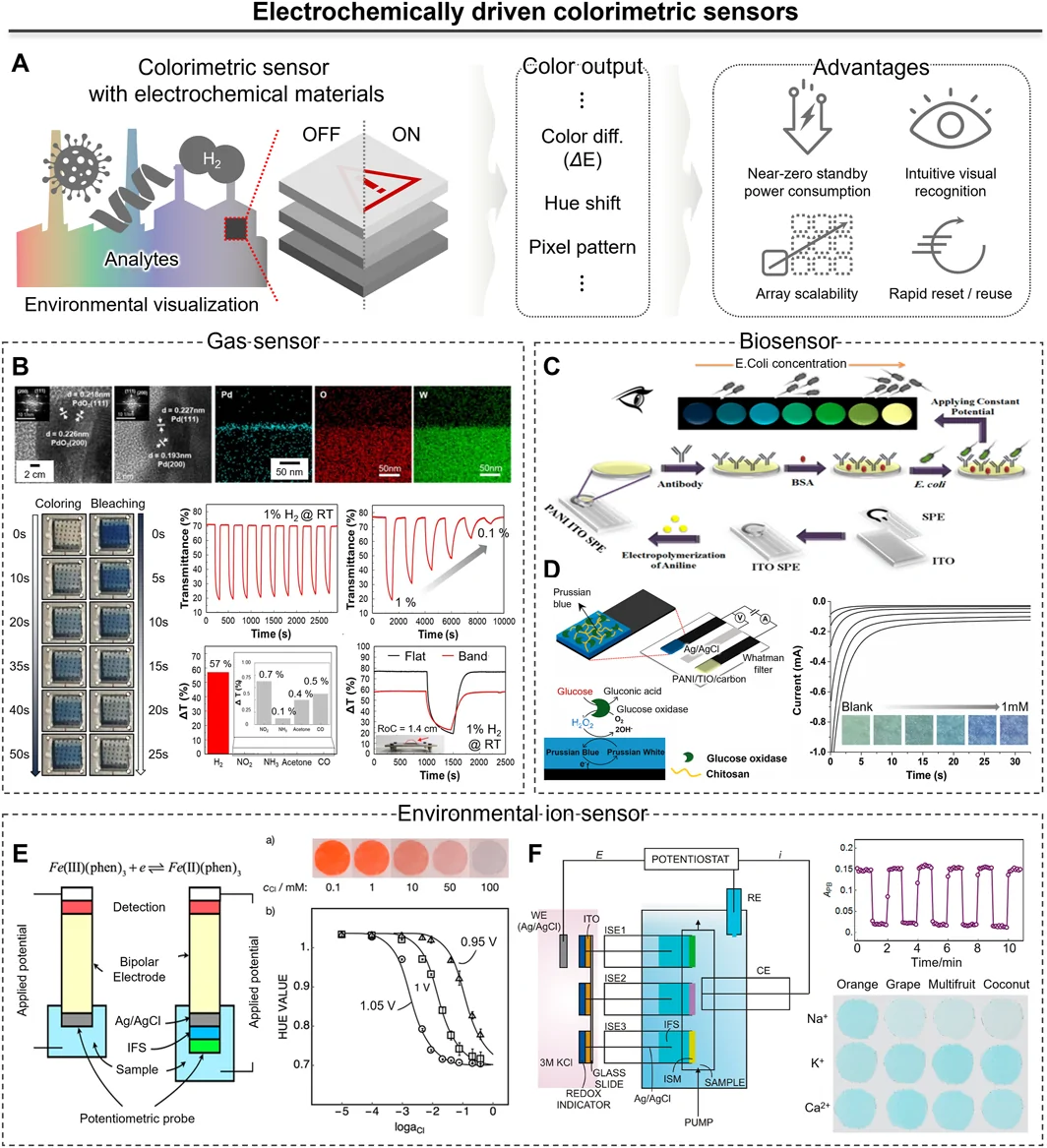
Electrochemical colorimetric sensors. (A) Outputs and advantages of electrochemical colorimetric sensing with analyte‐triggered on/off color. (B) EC film array bleaching/coloring under target gas (top) and reversible transients and calibration curves (bottom). Reproduced with permission [34]. Copyright 2022, Elsevier. (C) Electrochromic bioassay with recognition layers and electrodes (top) and concentration‐dependent color response with the corresponding LoD (bottom). Reproduced with permission [28]. Copyright 2019, American Chemical Society. (D) Microelectrode EC platform for sequence/enzyme readout (top) and specific, multiplexable patterns and spectra (bottom). Reproduced with permission [29]. Copyright 2022, Elsevier. (E) Potentiometric/ion‐exchange EC cell for ion colorimetry (top) and monotonic titration curve with color scale (bottom). Reproduced with permission [35]. Copyright 2018, American Chemical Society. (F) Multiplexed EC microcells driven by pulsed protocols (top) and repeatable ion‐specific color maps (bottom). Reproduced with permission [36]. Copyright 2020, American Chemical Society.

**FIGURE 7:**
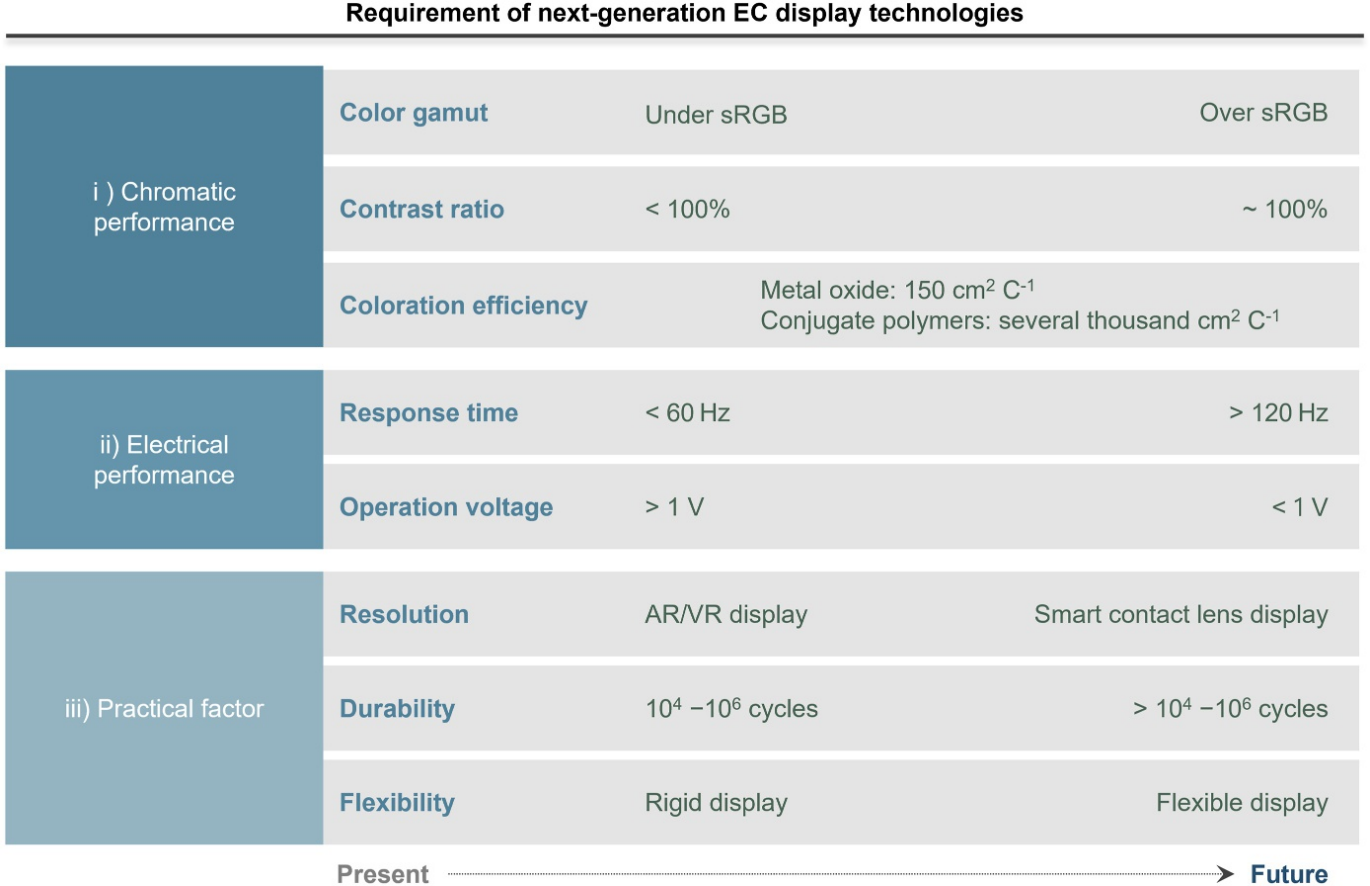
Outlook. Requirements for next‐generation EC displays—summary of chromatic, electrical, and practical targets showing the shift from present to future. Progress toward wide‐gamut, high‐coloration‐efficient color, faster low‐voltage switching, and durable, flexible form factors for wearable micro‐optics.

In the absence of external power or auxiliary instrumentation, the intrinsic chromatic response of sensing materials can be directly perceived to identify target gases—an ultralow‐power alternative to conventional high‐power gas‐sensing platforms [91]. To emphasize device‐level feasibility, we consider gas‐interactive electrochemical materials wherein analyte exposure reconfigures composition, redox state, or doping level, thereby producing a bias‐free colorimetric readout. Our analysis foregrounds material‐intrinsic transformation pathways, including ion/electron insertion, valence change, acid–base doping/de‐doping, and charge transfer processes, rather than electrically driven switching. As a representative bias‐free implementation, Cho et al. reported Pd‐nanoparticle‐decorated amorphous WO_3_ (a‐WO_3_) thin films in which catalytic H_2_ dissociation at Pd followed by rapid H^+^ insertion yields a reversible, visually salient color change (Figure 6B, top) [34, 92, 93]. E‐beam‐evaporated a‐WO_3_ affords high H^+^ diffusivity, enabling response/recovery dynamics unattainable with crystalline WO_3_. Time traces at 1% H_2_ (RT) show fast, highly reversible cycling, and the sensor delivers ∼57% optical modulation with ∼16 s recovery, exhibits strong selectivity (negligible response to NO_2_, NH_3_, acetone, CO), and retains performance under bending (Figure 6B, bottom).

Although bias‐free gas colorimetry highlights material‐intrinsic transduction, electrochemically driven colorimetry is equally compelling for biosensing at the point of care. From a biosensing perspective, laboratory‐based assays—culture/counting, enzyme‐linked immunosorbent assay (ELISA), and polymerase chain reaction (PCR)—offer high sensitivity and specificity but are slow, multistep, and equipment‐intensive, limiting utility when rapid decisions are required [94, 95, 96]. In contrast, point‐of‐care formats, such as lateral flow immunoassays (LFIA) and standalone electrochemical sensors, are portable and fast but fall short in sensitivity, specificity, and quantitative robustness [97, 98, 99]. Electrochromic colorimetric sensors address this gap by marrying a simple optical readout with the analytical rigor of electrochemical transduction [100, 101]. Ranjbar et al. introduced a visual E. coli assay that converts electrochemical signals into an optical readout (Figure 6C) [28]. PANI is electropolymerized on screen‐printed indium tin oxide (ITO) to provide (i) an electrochromic transducer under constant bias and (ii) an amine‐rich matrix for facile antibody immobilization. Upon target capture, the interfacial resistance increases, perturbing the PANI redox state and yielding a distinct color change at the electrode. The sensor covers ∼4 orders of magnitude in dynamic range with limits of detection (LoD) of ∼10^2^ CFU mL^−1^ by the naked eye and ∼10^1^ CFU mL^−1^ via image analysis. By coupling electrochemical sensitivity with a simple, instrument‐light optical readout, this approach enables low‐power, array‐scalable biosensing suited to water, food, and environmental monitoring. Unlike the antibody‐mediated, impedance‐coupled electrochromic assay on ITO‐SPE, Yeon et al. targeted small‐molecule glucose on a paper platform using an enzyme‐driven electrochromic readout (Figure 6D, left) [29, 100]. A paper‐based electrochemical colorimetric sensor is fabricated by screen‐printing carbon/graphite for WE and CE and Ag/AgCl for the reference electrode. One carbon electrode is functionalized with Prussian blue and glucose oxidase to transduce H_2_O_2_/glucose, whereas the opposing carbon electrode—used as the counter—is coated sequentially with ITO nanoparticles and PANI to provide a clear electrochromic color readout. Electrochemical operation mitigates traditional colorimetry drawbacks, improving response time, reusability, and color uniformity; quantitatively, the device achieves a ∼30 s response and a glucose LoD of 126 μM, with straightforward visual quantitation (Figure 6D, right). By combining the portability and simplicity of paper microfluidics with the analytical control of electrochemistry and the detector‐free visibility of PANI electrochromic materials, the sensor is well suited for point‐of‐care deployment, especially when paired with portable power sources.

Environmental ion sensing commonly relies on ion‐selective potentiometry, which is robust but typically requires wired readout and benchtop instrumentation. Closed bipolar electrode (C‐BPE) schemes address this limitation by transducing the potentiometric signal into an electrochromic reporter, producing a camera‐readable color change without additional spectrometers. This format preserves the selectivity of ion‐selective membranes while adding array compatibility, multiplexing, and straightforward visual quantitation. We highlight two representative demonstrations that establish the approach and extend it to arrayed environmental ion analysis. Jansod et al. developed a general colorimetric readout for potentiometric sensing using a C‐BPE architecture (Figure 6E, left) [35]. The ion‐selective element (e.g., Ag/AgCl for Cl^−^, a liquid‐membrane Ca^2+^‐selective electrode) is confined to one pole of the bipolar electrode, whereas an optically active reporter layer (ferroin) resides at the opposite pole—physically separating the sample compartment from the detection compartment. A potential imposed by solution contact drives the C‐BPE such that ion‐activity‐dependent potential changes at the sensing side modulate the detection‐side potential, turning over the reporter redox couple until a new equilibrium is reached and yielding a tunable color output (Figure 6E, right). The concept is material‐agnostic (extendable to other ion‐selective phases and reporter chemistries) and inherently low power, instrument‐light, and array‐compatible, providing a scalable route to camera‐readable potentiometric analysis within our framework. Building on this principle, the approach was extended to a simultaneous optical readout of ion‐selective electrode (ISE) arrays by integrating Prussian blue electrochromic films into multiple closed bipolar electrodes (Figure 6F, left) [36]. Each Prussian blue pixel, wired to a PVC‐membrane ISE (e.g., K^+^, Na^+^, Ca^2+^), undergoes potential‐dependent Prussian blue‐to‐Prussian white turnover, yielding a near‐Nernstian colorimetric response that is captured in parallel by camera imaging and quantified digitally. A single potentiostat maintains the global cell bias, whereas each pixel reports its connected probe's potential, enabling multiplexed, quantitative ion mapping even in strongly colored matrices (Figure 6F, right). Compared to ferroin‐based reporters, the Prussian blue film offers improved reproducibility and stability; mass transport is not rate‐limiting, and the measuring range is tunable via potential control—advancing the platform toward low‐power, array‐compatible environmental ion analysis within our evaluation framework.

## 4 Perspective (Outlook)

Electrochromic (EC) displays, in contrast to conventional emissive displays, operate based on modulation of light absorption and reflection, offering intrinsic advantages including superior energy efficiency, enhanced visibility under ambient illumination, and improved visual comfort for prolonged use (Figure 7). Achieving pure coloration is a critical requirement for high‐performance electrochromic displays. Unlike emissive displays that generate light at a single wavelength, electrochromic materials rely on intrinsic absorption and scattering, which often hinder the production of vivid and saturated colors. This limitation becomes more pronounced in subpixel architectures, where color purity and brightness are further reduced. The restricted optical modulation range of most electrochromic systems also makes it challenging to realize full‐color operation within a single device. Overcoming these constraints requires optimized photonic architectures and enhanced light–matter interactions to achieve efficient spectral control [102]. For future display technologies, the development of low‐power single pixel systems capable of producing high‐purity full‐color output is essential. Recent research has explored complementary designs that integrate both cathodic and anodic electrochromic materials, enabling energy‐efficient optical modulation [67]. Ultimately, achieving broad color tunability extending beyond the sRGB gamut is crucial for advancing next‐generation electrochromic display devices.

Optical contrast is a key parameter for evaluating the color‐switching performance of electrochromic devices, ensuring accurate and visually clear image presentation. It is generally quantified by the change in transmittance or reflectance at a specific wavelength. Depending on the device configuration, optical modulation is assessed by variations in transmittance for transmissive devices or reflectance for reflective devices. Furthermore, in electrochromic displays, achieving a wide optical modulation range and high contrast ratio is essential, as these factors not only determine color fidelity but also enable grayscale representation, which is critical for high‐quality image rendering in advanced display technologies. Additionally, coloration efficiency is a key parameter for evaluating electrochromic materials, representing the degree of optical absorbance or color change per unit charge consumed per unit area, defined as ΔA/Q. Here, ΔA denotes the change in absorbance at a specific wavelength, and Q represents the charge density. A higher coloration efficiency indicates stronger optical modulation with lower energy consumption, serving as a fundamental criterion for low‐power electrochromic operation. Consequently, coloration efficiency has long been regarded as a primary indicator of the coloration capability of electrochromic systems. The ability to achieve low‐voltage operation is particularly important for portable electronic platforms, including wearable devices and AR/VR systems, where energy efficiency is essential [103].

The electrical performance of electrochromic devices is defined by their ability to achieve low‐power and high‐speed switching under practical operating conditions. The response time is typically characterized as the duration required to reach 90% of the target optical modulation (ΔT or ΔR) during coloration or bleaching. Human vision perceives motion as continuous above 20 Hz and as smooth near 50 Hz, meaning that stable image visibility requires fast switching and sufficient optical contrast at high driving frequencies. To meet a 60‐Hz refresh rate, electrochromic displays therefore require response times below 16.7 ms [18, 104]. Organic electrochromic materials, typically composed of polymer chain structures, benefit from short ion and electron diffusion paths and structural flexibility, enabling relatively fast response times. Nevertheless, repeated redox cycling can induce polymer chain degradation and over‐oxidation, leading to compromised long‐term stability. In contrast, inorganic and metal oxide electrochromic materials exhibit robust structural stability, allowing reliable operation over tens of thousands of cycles, but their switching speed is generally limited by slow ion diffusion in the solid phase. From a materials and device design perspective, improvements can be achieved by shortening ion diffusion paths (e.g., through thinner or porous active layers), enhancing ionic conductivity of electrolytes, and reducing sheet resistance via optimized transparent electrodes and auxiliary bus‐line architectures. Additionally, pulse and duty‐cycle optimization helps balance the trade‐offs between switching speed, operating voltage, and device lifetime. The operating voltage directly influences both energy efficiency and circuit complexity, with an ideal electrochemical window below 1–2 V [18, 77, 105]. Low‐voltage operation is crucial for minimizing display power consumption and for realizing energy‐efficient next‐generation electrochromic display technologies.

Translating the foregoing optical and electrical merits into deployable platforms requires addressing three practical factors: resolution, durability, and flexibility. Resolution is anchored to current AR/VR display needs, demanding high‐density addressability across the field of view and high contrast/color purity under low‐voltage drive. Looking ahead to smart contact‐lens displays, ultrasmall, conformal metapixels that accommodate pupil size and ocular curvature, together with low‐cross‐talk wiring, become essential. Durability spans cycle life (repetitive switching), state retention, and environmental reliability; at present, stable operation in the 10^4^–10^6^‐cycle range with adequate retention is expected, whereas next‐generation systems should exceed these figures and simultaneously ensure reversibility and suppressed degradation [18]. In parallel, robustness under harsh conditions—temperature −40°C to 80°C, relative humidity ≤ 80%, and mechanical loading—must be maintained. For organic systems, mitigation of UV/oxygen‐induced photodegradation via encapsulation, UV‐blocking layers, and electrolyte/interfacial stabilization is critical. Flexibility has been demonstrated on both glass and polymer‐film substrates, yet translation to curved and variable‐curvature form factors at application grade will hinge on low‐temperature processing, interfacial/electrolyte stabilization, preservation of optical performance under repeated bending/torsion, and uniformity over large‐areas. Achieving these key performance metrics necessitates the integration of organic and inorganic active materials through photonic designs that effectively combine their complementary properties. In summary, co‐optimized, coexistent designs that balance the chromatic–electrical–practical axes are the key to advancing electrochromic displays in performance, reliability, and scalability, thereby enabling high‐resolution visualization, security patterning, and wearable optical interfaces.

## Author Contributions

**Hyo Eun Jeong:** writing – original draft, visualization. **Ji‐Eun Yeo:** writing – original draft, visualization. **Hyeon‐Ho Jeong:** writing – review and editing, project administration. **Young Min Song:** writing – review and editing, project administration, supervision.

## Funding

This work was supported by the Technology Innovation Program (RS‐2024‐00467230, Development of a Digital Healthcare Device for the Non‐invasive Continuous Monitoring of Myocardial Infarction Biomarkers Based on Mid‐Infrared Nano‐Optical Filters) funded by the Ministry of Trade, Industry and Energy (MOTIE, Korea). This work was also supported by the National Research Foundation of Korea (NRF) grant funded by the Korean government (MSIT) (RS‐2025‐16063568, RS‐2025‐16902996). This work was supported by the InnoCore program of the Ministry of Science and ICT (GIST InnoCore KH0830).

## Ethics Statement

The authors have nothing to report.

## Consent

Informed consent was obtained from all individuals included in this study.

## Conflicts of Interest

The authors declare no conflicts of interest.

## Data Availability

The data that support the findings of this study are available from the corresponding author upon reasonable request.
